# Gender differences in exercise efficiency: the influence of adiposity during low-intensity cycling in healthy Lebanese university students

**DOI:** 10.1186/s40101-025-00389-4

**Published:** 2025-03-12

**Authors:** Elie-Jacques Fares, Rédina Berkachy, Sarah Zaki

**Affiliations:** 1 Department of Nutrition and Food Sciences, Faculty of Agricultural and Food Sciences, American University of Beirut, Beirut, Lebanon; 2 Department of Medicine, Faculty of Medicine and Health Sciences, University of Sherbrooke, Sherbrooke, Canada; 3 School of Engineering and Architecture of Fribourg (HEIA-FR), University of Applied Sciences of Western Switzerland (HES-SO), Fribourg, Switzerland; 4 Faculty of Communication, Culture and Society, Università Della Svizzera Italiana (USI), Lugano, Switzerland

**Keywords:** Low-intensity exercise, Exercise efficiency, Adiposity, Gender differences, Cycling

## Abstract

**Introduction:**

Low-intensity physical activity plays a key role in weight regulation, and reduced engagement in such activities is associated with rising obesity rates. This study explored the relationship between body fat distribution and exercise efficiency during low-intensity cycling, comparable to everyday life, focusing on adiposity in men and women.

**Methods:**

Thirty participants (50% women and 50% men) underwent basal metabolic rate (BMR) measurements after an overnight fast. Following 500 ml water intake, they cycled at 60 rpm for 5 min at four intensities (20 W, 40 W, 60 W, 80 W), with respiratory parameters (i.e., energy expenditure (EE)) recorded using an indirect calorimeter system. Spearman correlations were used to assess the relationships among BMI, total body and trunk fat percentages, and delta efficiency (DE), which quantifies the energy cost associated with incremental work output during exercise.

**Results:**

A linear increase in EE with increasing power output was observed in both men and women, with men showing a slightly higher EE across all power levels. The linear regression equations for power between 20 and 80 W were highly predictive, with *R*^2^ values of 0.999 for men and 0.995 for women. Additionally, significant positive correlations were observed between BMI, fat percentage, trunk and limb fat percentages, and delta efficiency (DE) in women, explaining 45.7%, 34.7%, 34.1%, and 29.7% of the variance in DE, respectively. No significant correlations were found between these variables in men.

**Conclusion:**

This study demonstrated that body fat distribution, particularly in women, is significantly associated with exercise efficiency during low-intensity cycling. These findings highlight the need for larger studies that incorporate gender-specific considerations in exercise and targeted interventions.

## Introduction

Differences in energy metabolism efficiency are frequently linked to an individual’s tendency toward leanness or obesity [1–3]. This variation in metabolic efficiency can be studied by assessing energy expenditure (EE) under controlled conditions, either in a fasting (post-absorptive) state to measure basal metabolic rate [4], following food intake to evaluate the thermic effect of food [5, 6] or during dynamic exercise [7].

Low-intensity physical activity plays a significant role in weight regulation [8]. The replacement of these activities with sedentary behaviors, such as prolonged sitting or lying awake, has been shown to contribute more substantially to the increase in obesity and cardiometabolic diseases than moderate-to-vigorous leisure-time activities [9–12]. These insights have sparked growing interest in better monitoring, understanding, and promoting low-intensity physical activities in everyday life [8, 13–15].

In Lebanon, the prevalence of obesity was 14.5% and 18.8% for men and women, respectively [16]. Therefore, understanding the role of adiposity and fat distribution in energy expenditure phenotyping during low- to very-low-intensity activities can provide valuable insights for exercise prescription dedicated toward cardiovascular health or weight management.

Studies have shown that women generally possess a higher proportion of type I (oxidative) muscle fibers (27–35% greater in women) [17], particularly in muscles such as the vastus lateralis, and a smaller cross-sectional area of type II (glycolytic) fibers compared to men. This muscle composition enables women to maintain a more stable cellular energy balance during prolonged submaximal exercise at the same relative workload, as evidenced by a higher AMP/ATP ratio and smaller ATP reductions in women during prolonged or repeated high-intensity exercise [17].

The higher capillary density in women’s muscles further supports improved oxidative substrate utilization, leading to greater fat oxidation during submaximal exercise [18].

Fares et al. [7] examined energy expenditure phenotypes in inactive Caucasian men and women using low-power cycling and found that variations in body composition did not influence cycling efficiency. However, their study did not explore the specific effect of ethnicity and its relationship with body fat distribution on delta efficiency. Delta efficiency is a measure of the energy cost associated with incremental work output during exercise. It is calculated as the reciprocal of the slope of the linear relationship between EE and mechanical power. Unlike other measures, such as gross efficiency or net efficiency, which include resting or baseline EE in their calculations, delta efficiency does not rely on the assumption that baseline EE remains constant during exercise. This distinction is important because increases in exercise intensity inevitably elevate the energy demands for cardiorespiratory functions and contracting skeletal muscles. As highlighted by Fares et al., delta efficiency provides a robust and reproducible estimate of muscle efficiency with minimal variability, making it a reliable metric for assessing energy dynamics during physical activity [7].

The aim of this study was to investigate the relationship between body fat distribution, exercise (delta) efficiency, and gender in Lebanese university students.

Based on the existing literature and gaps in knowledge, the following hypotheses were proposed:

- Body fat distribution is related to delta efficiency during low-intensity cycling in Lebanese university students.
- Gender will moderate the relationship between body fat distribution and delta efficiency in low-intensity cycling.

## Materials and methods

### Participants

A convenient sample of 30 participants (50% women, 50% men) is included in this cross-sectional study (see Table 1), aged 18–35 years with a BMI of 24.4 ± 6.1 kg/m^2^, with low caffeine and tea consumption, nonsmoking, and moderate or no alcohol intake. The exclusion criteria included physical limitations, cardiovascular disease, pregnancy, unstable body weight, and active weight loss. Participants signed informed consent forms and completed anthropometric (i.e., height) and body composition assessments (i.e., fat and fat-free mass) using a bioelectrical impedance analysis device (InBody 770, InBody Co., Ltd., Seoul, Korea). Participants were instructed to follow an overnight 12-h fast, avoid intense physical activity and refrain from consuming caffeine or alcohol for 24 h before the test. Participants were asked to remove any clothing that might affect the measurement (i.e., heavy clothing, jewelry) and to stand barefoot on the foot electrodes. They were also asked to grip the hand electrodes to ensure proper contact with the device. They were instructed to stand upright with their arms slightly away from their body and to avoid any movement during the measurement.

**Table 1:** Characteristics of the study participants, presented as mean (± SD) for men (*n* = 15) and women (*n* = 15). Significant differences at 5% between men and women are observed in weight, BMI, fat mass percentage, fat mass (kg), trunk fat percentage, lower limb fat percentage, and basal metabolic rate, with *P < 0.05 indicating statistically significant differences between the two groups

**Participant characteristics**			
	**Men (15)**	**Women (15)**	*p*-value of the difference between men and women
**Age (years)**	21.1 (4.2)	21.6 (3.4)	***p* > 0.05**
**Weight (kg)**	80.6 (20.4)	60.1 (11.7)	*p* = 0.014*
**Height (m)**	176.5 (7.2)	162.1 (4.0)	***p* > 0.05**
**BMI (kg/m**^**2**^**)**	26.1 (7.4)	22.8 (4.1)	*p* = 0.015*
**Fat mass (%)**	21.9 (13.0)	32.3 (7.3)	*p* = 0.031*
**Fat mass (kg)**	19.9 (16.4)	20.1(8.5)	*p* = 0.021*
**Fat-free mass (kg)**	60.7 (6.6)	40.0 (4.1)	***p* > 0.05**
**Trunk fat (%)**	23.4 (14.0)	34.2 (7.5)	*p* = 0.015*
**Lower limb fat (%)**	21.4 (11.4)	33.1 (6.8)	*p* = 0.002*
**Basal metabolic rate (Kcal/min)**	1.423 (0.267)	1.221 (0.129)	*p* = 0.041*

Values are mean (± SD), *p* < 0.05*

### Protocol

**Fig. 1:**
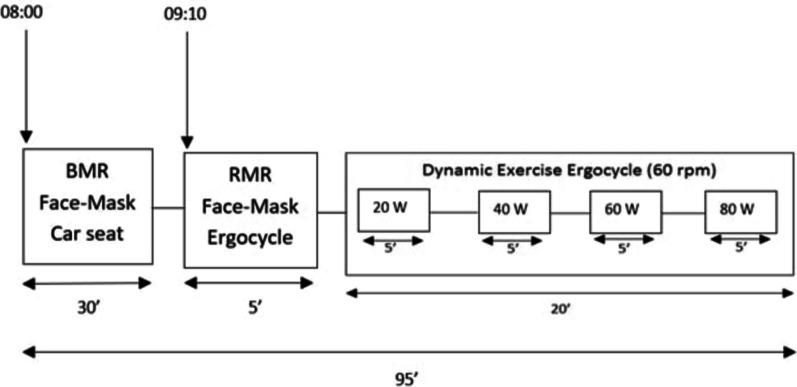
Testing protocol. BMR, basal metabolic rate; RMR, resting metabolic rate; rpm, revolutions per minute

After an overnight fast, participants came to the lab, had their basal metabolic rate measured, and then had 500 ml of water. Thirty-five minutes later, they started pedaling the ergocycle. They pedaled at 60 rpm for 5 min at each of the following intensities (20 Watts (W), 40 W, 60 W, 80 W). Female participants were tested during the follicular phase of the ovarian cycle. Respiratory parameters were analyzed using an ergometer (E100, ergo, COSMED, Rome, Italy) and a Quark CPET (Cardiopulmonary Exercise Testing for gas exchange analysis VO2, VCO2 during exercise or resting) (COSMED®, Italy) connected to a sophisticated high-end silicone facemask (Fig. 1). Calibration was performed according to the manufacturer’s instructions before each test. The study was funded by the University Research Board at the American University of Beirut.

### Statistical analysis

All statistical analyses were performed using the Statistical Package for the Social Sciences (SPSS) software (version 23, IBM Corporation, Armonk, NY, USA), and figures were generated using Microsoft Excel (Office 365, version 2407) and R software (R version 4.3.3). Normality tests were performed for all the variables. In some cases, these tests reject the hypothesis of normality. For this reason, Spearman correlations were performed on all variables and linked to delta efficiency (DE). DE is calculated as the inverse of the slope of the linear relationship between energy expenditure (EE) and mechanical power. The variables used were BMI, total body fat percentage (FAT%), trunk fat percentage (trunk Fat%), and lower limb fat percentage (limb Fat%). *T*-tests were done to compare EE and RQ values between genders. For all tests, statistical significance was set at the 0.05 threshold (*p* < 0.05*) and at the 0.01 threshold (*p* < 0.01**). Because the normality assumption was not verified for all groups, bootstrap linear regressions were performed. On the other hand, the power analysis for the linear regression with a power of 0.8 and an expected correlation of 0.7 reveals a sample size of 12 participants, confirming the protocol experiment.

## Results

**Fig. 2:**
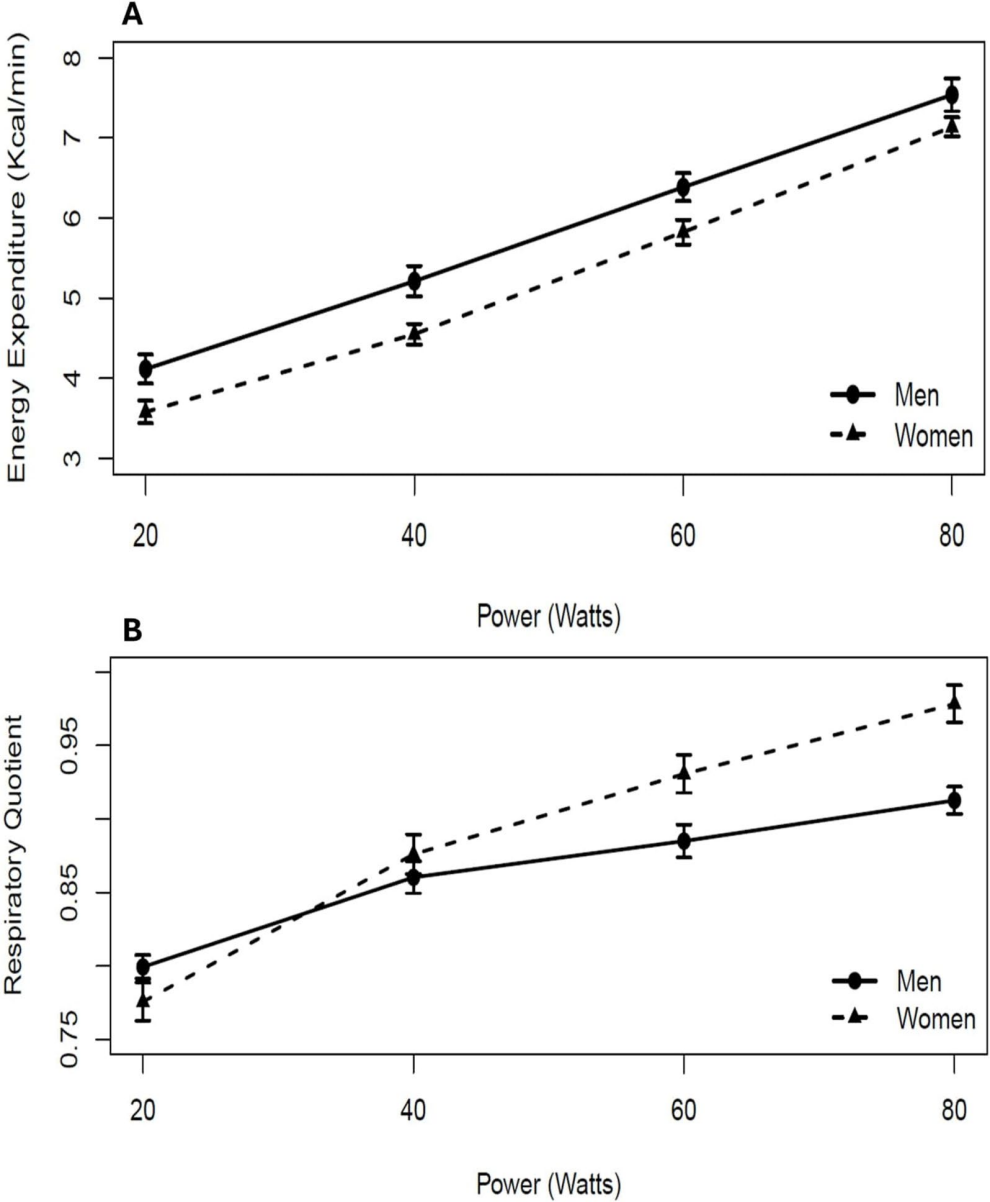
Effect of power on energy expenditure (**A**) and respiratory quotient (**B**) in both men (black dots, *n* = 15) and women (black triangles, *n* = 15). Values are mean ± SD

**Fig. 3:**
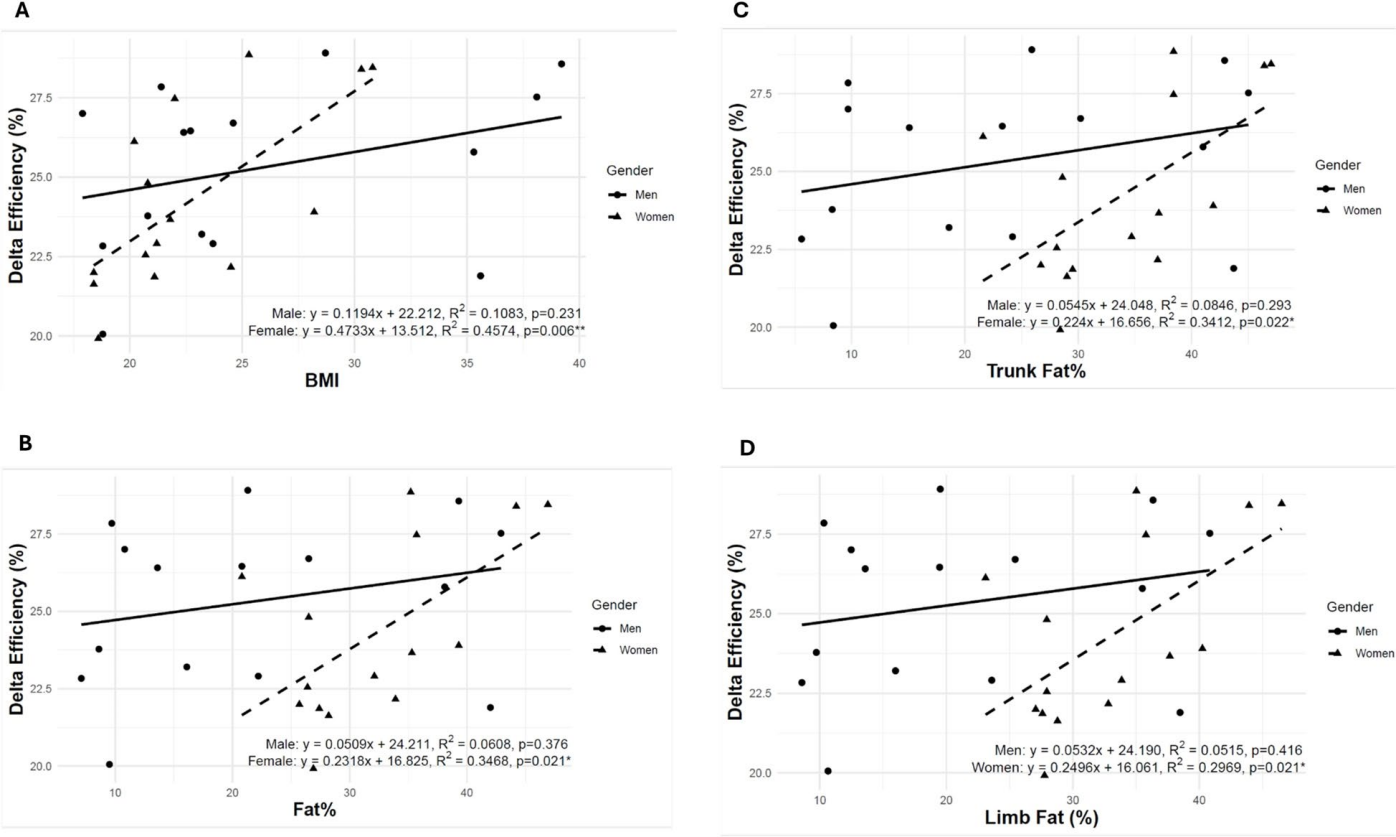
Linear regressions between delta efficiency (DE) (dependent variable) and adiposity measures (independent variables) during low-intensity cycling in men and women. **A** DE vs. BMI (*R*^2^ = 0.457 for women, *R*^2^ = 0.108 for men). **B** DE vs. total body fat percentage (*R*^2^ = 0.346 for women, *R*^2^ = 0.060 for men). **C** DE vs. trunk fat percentage (*R*^2^ = 0.341 for women, *R*^2^ = 0.084 for men). **D** DE vs. limb fat percentage (*R*^2^ = 0.297 for women, *R*.^2^ = 0.051 for men). Linear regression lines are shown for women (black triangles) and men (black dots)

Figure 2 demonstrates the relationship between energy expenditure (Kcal/min) (A), respiratory quotient (B), and power (Watts) in both men and women. It shows a linear increase in EE with increasing power output for both genders. Men exhibited slightly but significantly higher-energy expenditure than women at 20 (*p* = 0.0271), 40 (*p* = 0.0085), and 60 (*p* = 0.0225) W. Conversely, the respiratory quotient followed a similar trend with power output, but women significantly showed higher mean values at 60 (*p* = 0.0123) and 80 W (*p* = 0.0003) compared to men. Figure 3 shows the application of linear regression for each pair of variables: DE vs. BMI, DE vs. FAT%, DE vs. trunk Fat%, and DE vs limb Fat%. The black triangles represent women, and the black dots represent men. Bootstrap linear regression lines are shown for women (dotted line) and for men (straight line). Spearman correlation was calculated for each group.

The results in Fig. 3 indicate significant positive correlations among BMI (cor = 0.711, *p* = 0.003**), FAT% (cor = 0.521, *p* = 0.046*), trunk Fat% (cor = 0.588, *p* = 0.022*), limb Fat% (cor = 0.579, *p* = 0.024*), and DE in women. The regression slope is 0.4733 (*R*^2^ = 0.4574, *p* = 0.007** for the estimate), showing that 45.7% of the variance in DE was explained by BMI in women. Similarly, FAT% in women showed a positive correlation with DE, with a slope of 0.2318 (*R*^2^ = 0.3468, *p* = 0.029* for the estimate), indicating that 34.7% of the variance in DE was explained by FAT%. The trunk Fat% also demonstrated a positive correlation, with a slope of 0.224 (*R*^2^ = 0.3412, *p* = 0.058 for the estimate), explaining 34.1% of the variance in DE. Finally, the limb Fat% showed a similar correlation with a slope of 0.2496 (*R*^2^ = 0.2969, *p* = 0.021* for the estimate), explaining 29.69% of the variance in DE. In contrast, no correlations or linear regression models were significant in men for any of the variables. These findings suggest that body composition metrics are more strongly associated with DE in women than men.

## Discussion

The findings of this study confirmed significant relationships between body composition metrics and exercise efficiency, particularly among women. The observed positive correlations between BMI, FAT%, trunk Fat%, and limb Fat% with DE during low-intensity cycling highlight the differential impact of adiposity on metabolic function across genders.

Research has consistently shown that higher levels of body fat are associated with poorer physical performance and lower-energy efficiency, particularly during weight-bearing activities [19].

However, our study is distinct in its focus on low-intensity cycling, an activity that does not significantly burden the musculoskeletal system, yet reveals pronounced exercise efficiencies that might be linked to higher adiposity in women. These findings suggest that women’s metabolic responses to exercise may be more sensitive to body fat distribution than men’s, contrasting with prior research [7], who found no significant influence of body composition on cycling efficiency in a mixed-gender Caucasian sample.

The results of Chen et al. [20] further reinforced the complexity of the relationship between body fat and exercise efficiency. In their study, conducted on a heterogeneous population of healthy adults, they observed that walking efficiency decreased with increasing body fatness for both men and women at normal speeds (0.9 to 1.2 m/s), but not at the lowest speed (0.6 m/s) in men where the opposite occurred [20]. This finding aligns with our results, which indicate that higher fat percentages, particularly in the trunk and limbs, affect metabolic efficiency during low-intensity cycling. Chen et al. also noted significant between-subject variations in efficiency, which could be explained by differences in body composition, gender, and habituation to physical activity [18, 20]. Interestingly, they found no significant correlation between body fat and stepping efficiency, which is in contrast with our cycling results.

The stronger association between body fat distribution and exercise efficiency in women could be explained by differences in muscle fiber composition, hormone levels, and substrate utilization during exercise [18, 21]. Women typically have a higher percentage of body fat and a different fat distribution pattern, which might contribute to more efficient energy usage during low-intensity activities [21]. Unlike in women, no significant correlation was observed between exercise efficiency and body fat in men. This disparity may stem from physiological differences, such as a lower proportion of type I muscle fibers and reduced capillary density, which could impact energy utilization during exercise [17, 18]. In addition, the decreased efficiency observed in men with lower adiposity than women may be due to a greater reliance on fast-twitch muscle fibers, which require more oxygen per unit of work than slow-twitch fibers. This pattern may also involve increased recruitment of active motor units, which raises oxygen consumption relative to workload increments [18]. Furthermore, the role of estrogen in fat metabolism could influence these differences, as estrogen is known to enhance fat utilization, potentially leading to improved exercise efficiency [22]. Chen et al. [20] reported greater exercise efficiency in women during stepping exercises, attributing this to differences in body mass, height, and habitual activity. Another study on differences in body composition and lower extremity fat distribution found that extramyocellular lipid (EMCL) content was positively associated with five times sit-to-stand test performance in women, while no significant association was observed in men. This suggests that higher EMCL levels in the soleus and calf subcutaneous fat may support physical function in women, whereas intramyocellular lipid accumulation appears negatively linked to performance. These findings highlight the role of segmental fat distribution in muscular performance [23].

This study showed that EE increases linearly with power output in both men and women. Men consistently showed slightly higher EE across all power levels, likely because of their greater muscle mass and the associated higher energy demands during exercise. Despite this, women with higher body fat distribution demonstrated substantial exercise efficiency, as shown in Fig. 3.

These combined factors lead to a lower delta efficiency in men, as more motor units are activated to a smaller extent, effectively “protecting” them from high metabolic strain but increasing the overall oxygen cost of exercise. This suggests that women’s muscle composition (i.e., higher proportion of type I muscle fibers and capillary density in women) and recruitment patterns, with different body composition parameters, afford them greater efficiency at low-intensity activities, favoring oxidative pathways and maintaining energy balance during exercise [7, 18].

In regard to substrate oxidation, the RQ remained relatively stable at very low intensities (< 50 W), consistent with findings by Calonne et al. [24]; however, RQ began to increase between 40 and 50 W in both genders, with no significant differences observed at these intensities. This corresponds to the point where EE surpasses the typical 2–3 MET range, associated with common daily activities such as sitting, standing, walking, and stair climbing. In our study, similar trends were observed, but significant gender differences emerged at higher intensities (60 W and 80 W), suggesting potential gender-specific variations in metabolic response and substrate utilization at greater workloads.

The strengths of this study lie in its focus on gender-specific differences in body fat distribution and exercise efficiency during low-intensity activity using direct measurement tools such as the indirect calorimetry system to capture reliable data. Examining multiple exercise intensities provides valuable insights into physical activity in daily life. However, the small sample size, limited participant diversity, and one exercise type (dynamic) reduced the generalizability of the findings. Additionally, the lack of control for other influencing factors, such as diet or fitness level, limits the ability to establish causality. Further research with larger, more diverse samples, as well as standardized isometric exercises, as mentioned in Sarafian et al. [25], is needed to confirm these results.

## Conclusion

This study examined the distribution of body fat, with a particular focus on trunk and lower limb fat. It found that women with higher trunk or lower limb fat percentages demonstrated greater exercise efficiency during low-to-moderate intensity cycling compared to men. These findings underscore the importance of conducting larger studies that incorporate gender-specific factors in the analysis of exercise efficiency, visceral adiposity, and other fat distribution patterns.

## Data Availability

The data will be made available on request.
